# Role of biliary stent and neoadjuvant chemotherapy in the pancreatic tumor microbiome

**DOI:** 10.1186/s12866-021-02339-3

**Published:** 2021-10-16

**Authors:** Harika Nalluri, Eric Jensen, Christopher Staley

**Affiliations:** 1 Department of Surgery, University of Minnesota, Minneapolis, MN USA; 2 Masonic Cancer Center, University of Minnesota, Minneapolis, MN USA; 3 Biotechnology Institute, University of Minnesota, St. Paul, MN USA

**Keywords:** Microbiota, Pancreatic tumor microbiota, Pancreatic adenocarcinoma, Biliary stent, Neoadjuvant chemotherapy

## Abstract

**Background:**

Intra-tumor microbiota have been implicated in pancreatic ductal adenocarcinoma (PDAC) development, treatment response and post-treatment survivorship. Moreover, therapeutic interventions targeting microbiota may improve the response to chemotherapy and immunotherapy, further emphasizing the critical need to understand the origins of and growth of bacteria within the pancreatic tumor microenvironment. Here, we studied the role of several clinical factors on the bacterial colonization of PDAC.

**Results:**

We obtained matched tumor and normal pancreatic tissue specimens from 27 patients who had undergone surgical resection for PDAC between 2011 and 2015 from the University of Minnesota Biological Materials Procurement Network (BioNet). We found that 26 (48%) out of 54 pancreatic tissue samples harbored detectable bacterial communities using real-time PCR targeting the 16S rRNA gene. Bacterial colonization was detected significantly more frequently in samples from patients who had pancreatic head tumors, underwent Whipple procedure, or had preoperative biliary stent placement. There was also a significantly greater relative abundance of microbiota from the family *Enterobacteriaceae* among samples from patients who underwent biliary stent placement or neoadjuvant treatment with a combination of Gemcitabine and Paclitaxel.

**Conclusions:**

These findings suggest that biliary stent placement and neoadjuvant chemotherapy are associated with specific alterations that promote the infiltration and growth of intra-tumor bacteria in the setting of PDAC. Further studies exploring whether specific bacterial communities could contribute to increased chemoresistance will be essential for optimizing medical therapies in the future.

## Background

Pancreatic ductal adenocarcinoma (PDAC) has an average five-year survival of 9% across all stages and is the fourth leading cause of cancer death in men and women in the United States [1]. Fewer than 20% of patients present with resectable tumors, and most patients develop disease recurrence despite curative-intent surgery and adjuvant chemotherapy, with or without chemoradiation [2]. A lack of early diagnostic markers, rapid disease progression, and poor sensitivity to adjuvant therapies contribute to this dismal prognosis. Therefore, novel strategies to advance current therapeutic options are needed.

Once considered a sterile organ, recent evidence demonstrates that the pancreas harbors a microbiota, which is even more abundant in pancreatic cancer tissue [3]. The presence of intra-tumor microbiota in pancreatic cancer has many implications. The tumor microbiome has been linked to PDAC oncogenesis and cancer-associated inflammation [4]. Tumor microbial signatures may be indicative of the likelihood of PDAC survival, as a distinct bacterial composition with greater diversity has been found in tumors from long-term survivors (LTS) compared to short-term survivors (STS) [5]. In fact, transfer of gut microbiota from LTS induced an antitumor response in tumor-bearing mice that was not observed after transfer of microbiota from STS. In an orthotopic murine model of PDAC, gut bacterial ablation with antibiotics led to immunogenic reprogramming of the tumor microenvironment and improved the efficacy of immunotherapy [3]. Furthermore, a landmark study by Geller et al. revealed that intra-tumor *Gammaproteobacteria* can metabolize the chemotherapy agent Gemcitabine, suggesting a potential role in mediating chemoresistance in PDAC [6].

Bacterial colonization of pancreatic cancer tissue is thought to be due to the migration of microbiota from oral, gastrointestinal, and hepatobiliary sources [7–11]. Routes of colonization of intestinal bacteria are suspected to include the bloodstream, lymphatic system, and direct reflux from the duodenum [12]. Interventions such as preoperative biliary drainage and neoadjuvant chemotherapy have been shown to alter biliary and duodenal microbiota, and pancreatic head carcinomas have been strongly correlated with bactibilia [13–16]. Yet, the influence of these clinical factors on the infiltration and growth of bacteria within the pancreatic tumor microenvironment is poorly characterized. Geller et al. began to investigate this and found that patients who underwent pancreatic duct instrumentation had more bacteria in their pancreas tumors than those who had not undergone instrumentation [6]. However, further research to define the impact of these clinical factors on the bacterial colonization of pancreatic tumor tissue is imperative. In this study, we performed a retrospective, exploratory analysis of pancreatic tissue samples from patients who underwent surgical resection for PDAC. We hypothesized that clinical parameters such as biliary stent placement and neoadjuvant chemotherapy likely contribute to intra-tumor bacterial colonization.

## Results

### Clinical characteristics

Matched malignant pancreatic tissue and normal adjacent pancreatic tissue samples were obtained from 27 patients (*n* = 54 samples) who underwent surgical resection for PDAC at the University of Minnesota between 2011 and 2015. Patient demographic and clinical characteristics are presented in Table 1. This cohort consisted of 18 men (67%) and 9 women (33%) with a mean age of 64 years and mean body mass index (BMI) of 29 kg/m^2^. Tumors were most commonly located in the head of the pancreas (*n* = 18, 67%) and classified as stage IIB (*n* = 14, 52%). A preoperative biliary stent was placed in 16 (59%) patients, with similar frequency in choice of plastic or metal stent material. Neoadjuvant chemotherapy was administered in 10 (37%) patients, and choice of specific chemotherapy regimen was left to the discretion of the treating oncologist. Few patients had antibiotic exposures within 6 weeks pre-operatively (*n* = 3, 11%); one treated for cholangitis until 10 days prior to surgery, one treated for acute cholecystitis until 14 days prior to surgery, and one treated for acute diverticulitis until 5 weeks prior to surgery.

**Table 1:** Patient demographic and clinical characteristics

Number of patients, n	27
Male sex, n (%)	18 (67)
Age (years)	63.9 ± 10.4
BMI (kg/m^2^)	28.8 ± 4.3
Tumor stage, n (%)	
IA	0
IB	7 (26)
IIA	5 (18)
IIB	14 (52)
III	1 (4)
IV	0
Tumor Location, n (%)	
Head	18 (67)
Body	3 (11)
Tail	6 (22)
Preoperative biliary stent, n (%)	
No Stent	11 (41)
Stent	16 (59)
Stent type, n (%)	
Plastic	7 (26)
Metal	7 (26)
Unknown	2 (7)
Neoadjuvant chemotherapy, n (%)	
None	17 (63)
Gemcitabine	7 (26)
Gemcitabine/Paclitaxel	2 (7)
Gemcitabine/Oxaliplatin	1 (4)
Recent antibiotic exposure (< 6 weeks preoperatively)	
Yes	3 (11)
No	24 (89)
Operative procedure, n (%)	
Pancreaticoduodenectomy (Whipple)	18 (67)
Distal pancreatectomy and splenectomy	9 (33)
Perioperative antibiotic prophylaxis, n (%)	
Cefazolin	12 (44)
Cefoxitin	7 (26)
Cefotetan	5 (18)
Clindamycin	1 (4)
Levofloxacin	1 (4)
Unknown	1 (4)
Infectious postoperative complications, n (%)	
None	19 (70)
Superficial SSI	3 (11)
Deep/organ-space SSI	2 (7)
Pancreatic anastomotic leak	5 (18)
*Clostridium difficile* colitis	2 (7)
Urinary tract infection	2 (7)
Parotiditis	1 (4)
Survival, n (%)	
Short-term survival (< 5 years)	17 (63)
Long-term survival (> 5 years)	3 (11)
Lost to follow up	7 (26)

Continuous data presented as mean ± standard deviation. *BMI* Body mass index, *SSI* Surgical site infection

A majority of patients underwent Whipple procedure (*n* = 18, 67%). Choice of perioperative antibiotic prophylaxis was based on surgeon preference, and the majority of patients received either Cefazolin (*n* = 12, 44%), Cefoxitin (*n* = 7, 26%), or Cefotetan (*n* = 5, 18%). Levofloxacin (*n* = 1, 4%) and Clindamycin (*n* = 1, 4%) were given to patients with penicillin allergy. Infectious postoperative complications occurred in 8 (30%) patients. Most patients were short-term survivors who lived less than 5 years post-operatively (*n* = 17, 63%), and few were long-term survivors who lived greater than 5 years post-operatively (*n* = 3, 11%).

### Bacterial colonization associated with clinical features

We investigated whether pancreatic tissue samples harbored detectable bacterial DNA via real-time PCR targeting the bacterial 16S rRNA gene. Out of the 27 patients examined, bacteria were detected in both matched samples from 8 (29.6%) patients, only malignant tissue sample from 5 (18.5%) patients, only normal adjacent tissue sample from 5 (18.5%) patients, and neither matched sample in 9 (33.3%) patients. To understand which clinical parameters may contribute to the presence of microbiota on PDAC tumor tissue, we compared malignant pancreatic tissue samples with (*n* = 13, 48%) and without (*n* = 14, 52%) bacterial colonization at the time of surgery (Table 2).

**Table 2:** Comparison between malignant PDAC tissue samples with and without bacterial colonization

	Bacterial colonization (*n* = 13)	No bacterial colonization (*n* = 14)	*P* value
Male sex, n (%)	9 (69)	9 (64)	0.785
Age (years)	63.7 ± 9.5	64 ± 11.8	0.622
BMI (kg/m^2^)	28.7 ± 5.2	28.9 ± 3.6	0.934
Tumor stage, n (%)			
IA	0	0	0.434
IB	2 (15)	5 (36)	
IIA	2 (15)	3 (21)	
IIB	8 (62)	6 (43)	
III	1 (8)	0	
IV	0	0	
Tumor Location, n (%)			
Head	13 (100)	5 (36)	**0.002**
Body	0	3 (21)	
Tail	0	6 (43)	
Preoperative biliary stent, n (%)			
Stent	12 (92)	4 (29)	**0.001**
No Stent	1 (8)	10 (71)	
Stent type, n (%)			
Plastic	6 (46)	1 (7)	0.565
Metal	5 (38)	2 (14)	
Unknown	1 (8)	1 (7)	
Neoadjuvant chemotherapy, n (%)			
None	7 (54)	10 (71)	0.303
Gemcitabine	3 (23)	4 (29)	
Gemcitabine/Paclitaxel	2 (15)	0	
Gemcitabine/Oxaliplatin	1 (8)	0	
Operative procedure, n (%)			
Pancreaticoduodenectomy (Whipple)	13 (100)	5 (36)	**0.0004**
Distal pancreatectomy and splenectomy	0	9 (64)	
Perioperative antibiotic prophylaxis, n (%)			
Cefazolin	5 (38)	7 (50)	0.123
Cefoxitin	6 (46)	1 (7)	
Cefotetan	1 (8)	4 (29)	
Clindamycin	1 (8)	0	
Levofloxacin	0	1 (7)	
Unknown	0	1 (7)	
Infectious postoperative complications, n (%)			
None	8 (62)	11 (79)	0.433
Superficial SSI	1 (8)	2 (14)	
Deep/organ-space SSI	2 (15)	0	
Pancreatic anastomotic leak	3 (23)	2 (14)	
*Clostridium difficile* colitis	2 (15)	0	
Urinary tract infection	1 (8)	1 (7)	
Parotiditis	1 (8)	0	
Survival, n (%)			
Short-term survival (< 5 years)	7 (54)	10 (71)	0.616
Long-term survival (> 5 years)	3 (23)	0	
Lost to follow up	3 (23)	4 (29)	

Continuous data presented as mean ± standard deviation. Bold indicates statistical significance (*P* < 0.05). *BMI* Body mass index, *SSI* Surgical site infection

No significant differences in bacterial colonization were observed based on sex, age, BMI, tumor stage, or receipt of neoadjuvant chemotherapy. Bacterial colonization occurred more frequently in samples from patients with tumors located in the head of the pancreas (χ2 = 6.0, *P* = 0.002) and those that required surgical resection with Whipple procedure (χ2 = 3.8, *P* < 0.001) compared to samples without bacterial colonization. Samples with bacterial colonization were also significantly more likely to be from patients who underwent preoperative biliary stent placement (χ2 = 3.8, *P* = 0.001) in contrast to samples without bacterial colonization. Yet, no difference based on type of biliary stent (χ2 = 6.0, *P* = 0.565) was observed. Nor were there significant differences in bacterial colonization associated with type of perioperative antibiotic prophylaxis, postoperative infectious complications, or short- versus long-term survival. This analysis was also performed with normal adjacent tissue and no significant differences in bacterial colonization were identified based on these clinical factors.

### *Enterobacteriaceae* linked to stent placement and neoadjuvant chemotherapy

**Fig. 1:**
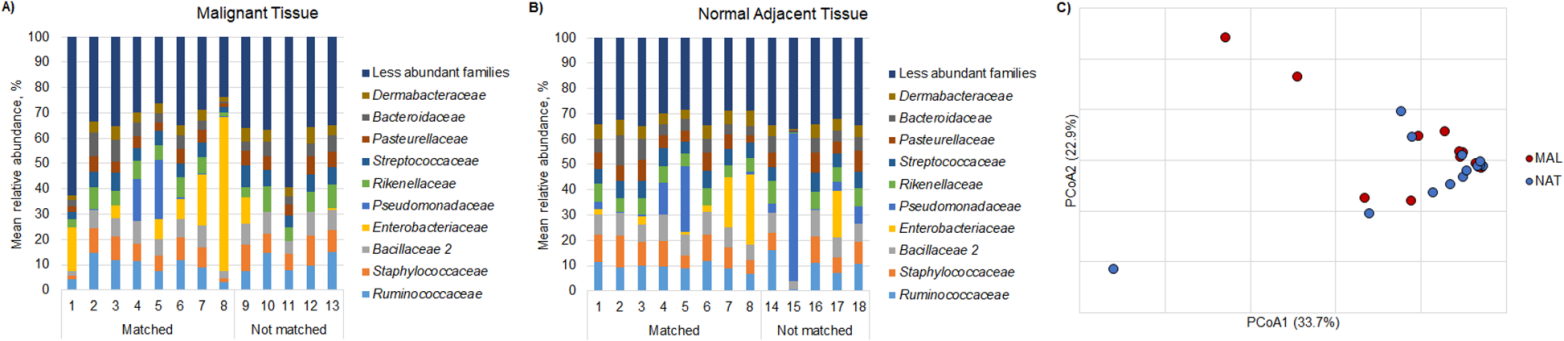
Microbiota composition in PDAC and normal tissue samples. Taxonomic profiles of predominant bacterial families by mean relative abundance (%) in **A** malignant tissue and **B** normal adjacent tissue samples. **C** Principal coordinate analysis of Bray-Curtis dissimilarities among malignant tissue (*MAL,* red) and normal adjacent tissue (*NAT,* blue) samples

Next, we performed 16S rRNA gene-based amplicon sequencing of the 26 samples that harbored bacterial signatures, as determined by real-time PCR, to characterize taxonomic profiles. Among both malignant and normal adjacent tissue samples, the most abundant families of bacteria included *Ruminococcaceae, Staphylococcaceae, Bacillaceae, Enterobacteriaceae*, and *Pseudomonadaceae* (Fig. 1A-B)*.* There were no significant differences in overall community composition between malignant and normal adjacent pancreatic tissue types (Fig. 1C, ANOSIM R = − 0.01, *P* = 0.60). There also were no significant differences in community composition based on receipt of preoperative biliary stent or neoadjuvant chemotherapy (R = 0.12 and 0.14, *P =* 0.23 and 0.13, respectively).

**Fig. 2:**
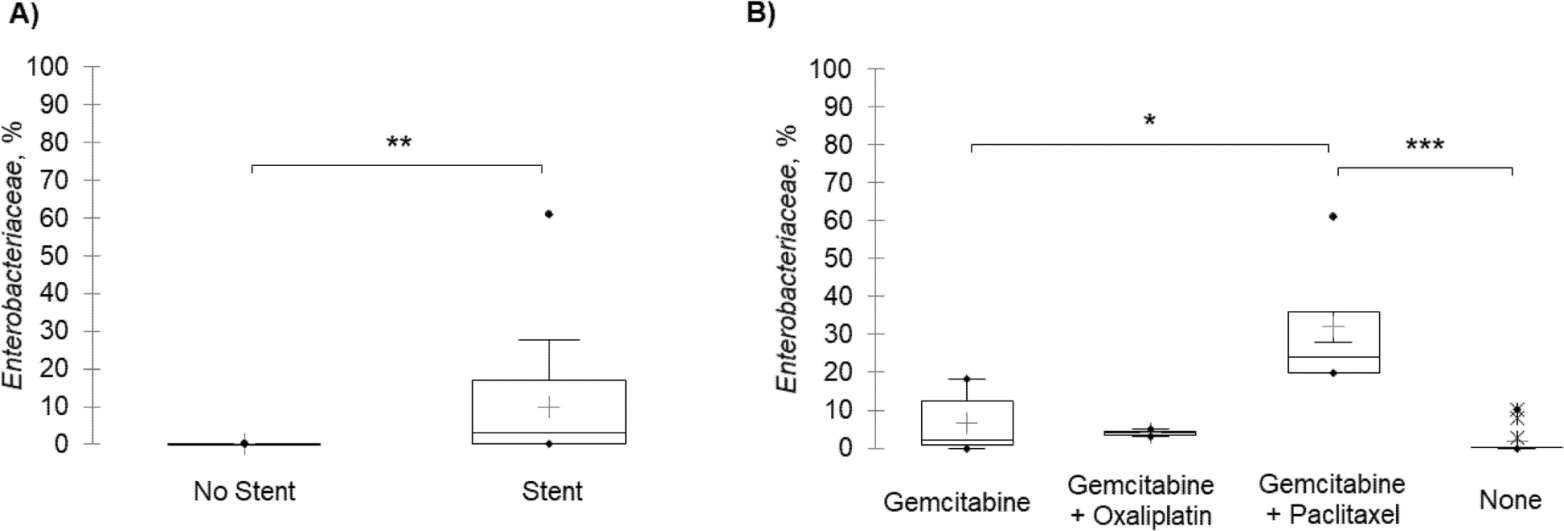
Comparison of mean relative abundance (%) of *Enterobacteriaceae* among samples from patients based on **A** placement of preoperative biliary stent and **B** receipt of neoadjuvant chemotherapy. *P ≤ 0.05, **P ≤ 0.01, ***P ≤ 0.001

Despite similar overall community composition, differences in the relative abundances of members of *Enterobacteriaceae*, a predominant family of microbiota among samples, were observed based on receipt of preoperative interventions (Fig. 2). There was significantly greater relative abundance of *Enterobacteriaceae* in samples from patients who underwent biliary stent placement (Dunn’s *post-hoc P* = 0.004). Additionally, samples from patients who underwent neoadjuvant treatment with a combination of Gemcitabine and Paclitaxel had significantly greater relative abundances of *Enterobacteriaceae* compared with samples from patients who underwent Gemcitabine therapy alone (*P* = 0.04), or patients who did not receive neoadjuvant chemotherapy at all (*P* < 0.001).

## Discussion

Resistance to antitumor drug therapies remains a significant challenge in the management of pancreatic cancer [17]. Tumor-associated microbiota have been implicated in reducing the efficacy of chemotherapeutics and could potentially serve as a novel target to optimize current medical therapies [7, 18]. In this study, we aimed to further understand the complex relationship between microbiota and PDAC by exploring clinical factors that may contribute to intra-tumor bacterial colonization.

Bacterial colonization of pancreatic tumor tissue was more likely in patients who had pancreatic head tumors and those that underwent Whipple procedure. This could be due to the fact that pancreatic head tumors, which often require Whipple procedure for resection, are closer in proximity to the gastrointestinal and hepatobiliary systems. Tumors at this location are also more likely to result in malignant biliary obstruction than distal tumors.

Intra-tumor microbial signatures were also more detectable in patients who had undergone preoperative biliary drainage with stent placement. Importantly, we found that biliary stent placement was correlated with greater relative abundances of *Enterobacteriaceae*, which include bacteria that have been postulated to confer resistance to the chemotherapeutic agent Gemcitabine [6]. As stated previously, Scheufele, et al. reported that stent placement affected the composition of biliary microbiota [15]. Migration of microbes from the biliary tract, adjacent to the pancreas, may influence the growth of intra-tumor microbiota. These investigators also postulated that plastic stents could lead to greater alterations in biliary microbiota because plastic stents have greater risk of recurrent biliary obstruction and poorer duration of patency compared to metal stents. Interestingly, we found that the type of biliary stent used did not impact the growth of intra-tumor bacteria.

We found that the multi-agent chemotherapy regimen comprised of Gemcitabine and Paclitaxel was also associated with greater relative abundances of *Enterobacteriaceae* compared to other chemotherapy regimens and no chemotherapy at all among our samples. This could be due to immunosuppression from the chemotherapy agent itself. Patients who undergo neoadjuvant therapy may additionally experience prolonged periods of stenting and increased exposure to antibiotics for stent-related cholangitis [13]. Future studies investigating the effects of neoadjuvant chemotherapy on PDAC microbiota should include multi-agent regimens as they may have variable outcomes.

Our findings limit definitive conclusions due to the retrospective fashion of data retrieval. Pancreatic tissue samples were difficult to obtain, and the small sample size of this study restricted the ability to perform well powered multivariate statistics. This may introduce bias from the lack of adjustment for potentially confounding factors, such as choice of preoperative antibiotic. We also did not have access to pancreatic ductal fluid samples or associated culture data, which may have provided additional insight into microbial changes in the tumor microenvironment. We chose to focus on family-level taxonomic assignment due to the uncertainty in classification at greater resolution due to low bacterial biomass. We did not explore the role of viral or fungal microbiota, which have also been implicated in PDAC oncogenesis [7, 19]. Additionally, the loss to follow-up of 26% of patients limits interpretation of findings related to survival. Nevertheless, the impact of several clinical factors in the growth of intra-tumor bacteria in PDAC observed in this study is novel. A better understanding of the role of intra-tumor microbiota in PDAC may aid in identifying new approaches in microbial modulation to optimize current chemotherapy options and improve patient outcomes.

## Conclusion

Preoperative biliary stent placement and neoadjuvant chemotherapy are associated with specific alterations in the pancreatic tumor microenvironment that may encourage bacterial colonization of PDAC tissue. Particularly, biliary stent placement and chemotherapy regimen of Gemcitabine and Paclitaxel may selectively promote the growth of *Enterobacteriaceae*, which have been postulated to contribute to chemoresistance. Future studies addressing whether these compositional alterations could contribute to increased chemoresistance may provide an opportunity to enhance the efficacy of medical therapies for pancreatic cancer.

## Methods

### Human sample collection

Matched tumor and normal adjacent pancreatic tissue samples were collected from 27 patients who had undergone surgical resection for PDAC between 2011 and 2015 from the University of Minnesota Biological Materials Procurement Network (BioNet). Surgical specimens were initially removed from patients intraoperatively by the surgeon and sent fresh to the pathologist. After gross examination, malignant and normal adjacent pancreatic tissue samples were sliced from the single surgical specimen. They were placed in clean petri dishes and given to BioNet, where they were frozen in liquid nitrogen in separate cryovials. Tissue handling by BioNet was performed in a clean environment, where there is possibility of contamination without complete sterility.

Normal adjacent pancreatic tissue was obtained from the site most distant from the tumor, with exact distance from the tumor depending on the size of the tumor and the entire specimen. Histologic quality control assessment was performed, and all normal adjacent tissue samples had 0% neoplastic tissue. Samples without histologic quality control assessment were excluded. Human specimens were obtained under approval by the Institutional Review Board (IRB). All specimens were stored at − 80 °C until further use.

### DNA extraction and real-time PCR

DNA was extracted from pancreatic tissue samples using the DNeasy PowerSoil kit (QIAGEN, Hilden, Germany) in accordance with manufacturer instructions. Total bacterial DNA in pancreatic tissue samples was determined by non-quantitative, real-time PCR. The V4 hypervariable region of the 16S rRNA gene was amplified using forward primer 515F (5′- GTGYCAGCMGCCGCGGTAA − 3′) and reverse primer 806R (5′- GGACTACNVGGGTWTCTAAT − 3′). Briefly, the reaction mix contained QuantiTect SYBR Green PCR Master Mix (QIAGEN), 0.5 μM forward and reverse primers, nuclease-free water, and sample DNA. PCR cycling conditions were: initial denaturation at 95 °C for 15 min, followed by 40 cycles of 30 s at 95 °C, 30 s at 50 °C, and 30 s at 72 °C. A standard curve using the V4 gene fragment cloned into a GenBlocks vector (IDT DNA, Coralville, IA) was run at concentrations from 5 × 10^6^ to 50 gene copies/reaction. Negative control (sterile water blank) reactions were also included. Reactions were run in triplicate on the LightCycler 480 Instrument II (Roche Diagnostics, Ltd., Basel, Switzerland). Due to low bacterial biomass and samples amplifying at the lower end of the standard curve, quantitative results were deemed unreliable, and samples were considered positive if they amplified before cycle 30 and had a melting temperature within the range of the standards (85.4 ± 0.7 °C).

### 16S rRNA gene-based amplicon sequencing

The V4 hypervariable region of the 16S rRNA gene was amplified using the 515F/806R primer set [20] by the University of Minnesota Genomics Center (UMGC, Minneapolis, MN, USA), as previously described [21]. Paired-end, dual indexed sequencing at read length of 300 nucleotides was performed on the Illumina MiSeq platform (Illumina, Inc., San Diego, CA, USA) by UMGC [21]. Negative sterile water controls were included in all sequencing runs and did not produce amplicons. Raw data are stored in the Sequence Read Archive under BioProject accession number SRP197553.

### Statistical analysis

Amplicon sequence data were processed and analyzed using mothur software ver 1.41.1 [22] with our previously published pipeline for quality screening and taxonomic annotation [23]. Reads were paired-end joined, quality trimmed, and aligned against the SILVA database ver. 132 [24]. Operational taxonomic units (OTUs) were binned at 97% similarity using the furthest-neighbor algorithm and taxonomic assignment was done against the Ribosomal Database Project ver. 16 [25]. A mean (± standard deviation) of 6866 ± 1677 reads per sample were obtained and samples were rarefied to 1800 reads for statistical comparisons, resulting in a mean estimated Good’s coverage of 97.1 ± 0.7%. After rarefaction, a mean of 162.6 ± 28.0 OTUs were observed among all samples.

Characteristics of samples with and without bacterial colonization were compared using Chi-squared analysis for categorical variables and Mann-Whitney-U test for continuous variables. Differences in overall community composition (beta diversity) were done using Bray-Curtis dissimilarities [26] and evaluated using analysis of similarity (ANOSIM) [27] with Bonferroni correction when appropriate using mothur. Samples were visualized by ordination using principal coordinate analysis (PCoA). Mean relative abundances of predominant families (found at mean abundances > 2.0%) were compared between groups with non-parametric Kruskal-Wallis rank sum test, followed by Bonferroni-corrected Dunn’s *post-hoc* test for pairwise comparisons. Analyses were conducted with XLSTAT (version 2020.2.3; Addinsoft, Belmont, MA, USA). All statistics were evaluated at α = 0.05.

## Data Availability

Raw data are stored in the Sequence Read Archive under BioProject accession number SRP197553.
